# Testis spectroscopy may predict sperm retrieval rate in men with non-obstructive azoospermia undergoing micro-TESE: A pilot study

**DOI:** 10.4274/jtgga.galenos.2019.2018.0154

**Published:** 2020-06-08

**Authors:** Önder Çelik, Şafak Hatırnaz, Aynur Erşahin, Alper Başbuğ, Gonca Y. Yıldırım, Vahit Özener, Neslihan Gürpınar, Sudenaz Çelik, Nilüfer Çelik, Tansu Küçük, Cihat Ünlü

**Affiliations:** 1 Department of Obstetrics and Gynecology, Private Office, Uşak, Turkey; 2 In Vitro Fertilization Unit, Medicana International Hospital, Samsun, Turkey; 3 In Vitro Fertilization Unit, Medicalpark Göztepe Hospital, İstanbul, Turkey; 4 Department of Obstetrics and Gynecology, Düzce University Faculty of Medicine, Düzce, Turkey; 5 Clinic of Obstetrics and Gynecology, University of Health Sciences, Kanuni Sultan Süleyman Training and Research Hospital, İstanbul, Turkey; 6 Department of Radiology, Barış Radiology Center, İzmir, Turkey; 7 Kent College High School, İzmir, Turkey; 8 Clinic of Biochemistry, University of Health Sciences, Behçet Uz Children’s Training and Research Hospital, İzmir, Turkey; 9 Department of Obstetrics and Gynecology, Acıbadem University Faculty of Medicine, İstanbul, Turkey

**Keywords:** Testis, magnetic resonance spectroscopy, sperm retrieval, micro-TESE, non-obstructive azoospermia

## Abstract

**Objective:**

To investigate whether prior testis magnetic resonance spectroscopy predicts the success or failure of micro-dissection testicular sperm extraction (micro-TESE) in patients with non-obstructive azoospermia (NOA).

**Material and Methods:**

Nine men with NOA who were scheduled for micro-TESE for the first time, 9 NOA men with a history of previous micro-TESE and 5 fertile men were enrolled. All NOA patients and fertile controls underwent testis spectroscopy. A multi-voxel spectroscopy sequence was used. Testicular signals of choline (Cho), creatine (Cr), myo-inositol (MI), lactate, and lipids were analyzed quantitatively and compared with the results of the micro-TESEs.

**Results:**

The most prominent peaks were Cho and Cr in the fertile controls and NOA subjects with positive sperm retrieval in the micro-TESE. A high Cho peak was detected in 87% of the NOA men with positive sperm retrieval. NOA men without sperm at the previous micro-TESE showed a marked decrease in Cho and Cr signals. For positive sperm retrieval in micro-TESE, the cut-off value of Cho was 1.46 ppm, the cut-off value of Cr was 1.43 ppm, and the cut-off value of MI was 0.79 ppm.

**Conclusion:**

Testis spectroscopy can be used as a non-invasive screening method to predict the success or failure of micro-TESE.

## Introduction

The fertility management of patients with non-obstructive azoospermia (NOA) involves micro-dissection testicular sperm extraction (micro-TESE) combined with intracytoplasmic sperm injection (ICSI) (1). Micro-TESE is not only a diagnostic tool for the presence of spermatozoa, but also a therapeutic procedure for retrieving sperm for ICSI. The sperm retrieval rate in men with NOA is reported to be 50% (2,3). However, micro-TESE is an invasive procedure that requires anesthesia. Moreover, repeated unsuccessful micro-TESE procedures can be devastating for fertility outcome. Concordantly, excessive and repeated tubule harvesting to retrieve spermatozoa may lead to complications, including testicular atrophy and hemorrhage, and a decline in serum androgen levels (4). In addition to being a surgically invasive procedure, there can be a severe psychological blow for infertile couples when sperm cannot be obtained during micro-TESE.

The development of non-invasive imaging techniques which can identify infertile men with NOA where a successful sperm retrieval outcome in micro-TESE can be expected is of great clinical significance. An evaluation of serum or seminal fluid biomarkers provides a minimally invasive diagnostic approach to predict the presence of spermatozoa in the testes of men with NOA. Allied to this, several predictors such as age, testicular volume, testicular histology, serum follicle stimulating hormone (FSH), inhibin and testosterone concentrations, and Y chromosome microdeletions have been used to test for the presence of spermatozoa in testicles (5,6,7,8,9). Nevertheless, each test has its own shortcomings and there are many examples of the limitations of these predictors, some of which are described below. Testicular biopsy and histology is the best predictor of micro-TESE outcome. However, it is not practical to perform a biopsy before micro-TESE, and recurrent surgery adds to the patient cost and increases the risk of complications. It has been reported that serum FSH levels indicate the status of seminiferous epithelium and can be used to predict spermatozoa status. A study conducted by Khelaia et al. (9) in 2015 reported that the sperm retrieval rate in NOA men with serum FSH levels between 10 and 15 mU/mL was 0%. Moreover, despite normal levels of circulating FSH, subjects may exhibit sperm maturation defects (10). Likewise, FSH levels show wide variations among infertile and fertile men (11). In spite of a strong positive correlation between testis volume and sperm retrieval rates, the calculation methods of testis volume are not standardized (12). In addition, despite normal testis volumes, subjects may show defects in spermatogenesis (10). While sperm recovery is possible in subjects with azoospermia factor c (AZFc) microdeletions, complete deletions in the AZFa or AZFb loci are not compatible with the presence of sperm (5,6).

In addition to biological predictors, some imaging techniques have been developed to predict the presence of spermatozoa in the testes of azoospermic men. Tsili et al. (13) assessed differences of apparent diffusion coefficient (ADC), fractional anisotropy (FA) and the association with the presence of spermatozoa after TESE. They reported that both ADC and FA are increased in NOA testes compared to age-matched controls. Multiphoton microscopy and Raman spectroscopy are further imaging techniques evaluating the testis and its content. However, each method requires either testis biopsy or biological fluid samples. In vitro techniques are also available (14). More importantly, DNA damage to sperm may occur if high laser intensity is used during these procedures. In short, globally accepted non-invasive biological or radiological tests that can predict the presence of spermatozoa in the testes of men with azoospermia undergoing micro-TESE have not been reported.

It is known that isolated regions of spermatogenic tissue may exist in the testicles of men with NOA (15). In the absence of non-invasive methods for the identification of these regions of spermatogenic tissue, invasive procedures such as testis biopsy and micro-TESE are the only diagnostic methods that are available to retrieve spermatozoa. Magnetic resonance spectroscopy (MRS) is a non-invasive imaging method that provides qualitative and quantitative information about the biochemical and molecular composition of living tissues, including testes. Any alteration in the molecular and cellular status of living tissues translate into signal intensity, which can be detected by MRS. Because each living tissue has a unique spectrum, spectral signal intensity or a chemical shift might predict the different in vivo pathological processes at a cellular level (16). The feasibility of MRS for evaluating female and male reproductive organs has been shown by our team and others (16,17,18). However, it remains to be determined whether spectroscopy of the testes before micro-TESE can predict the presence of sperm in harvested testis specimens. A comprehensive literature search did not reveal any studies investigating the predictor effects of testicular MRS in NOA men undergoing micro-TESE. The present study thus aimed to determine whether prior testis MRS can predict the success or failure of micro-TESE, as well its value in the management of NOA patients undergoing initial or repeat micro-TESE.

## Material and Methods

This pilot study was approved by the Ethical Commitee of Kanuni Sultan Süleyman Training and Research Hospital (approval number: KAEK/2017.1.13). In total, 18 men with NOA with a mean age of 37 (range: 27-48 years) and five fertile controls were included in the study. Azoospermia was defined as the absence of sperm cells in the seminal fluid. All patients were confirmed to be azoospermic through at least two semen analyses. Nine of the 18 patients had previously undergone micro-TESE, and these cases were evaluated retrospectively. Three patients were sperm positive on micro-TESE, but sperm was not found in the other six patients. Due to weak choline (Cho) and creatine (Cr) signals in their spectra, the six NOA men with negative micro-TESE anamnesis were not recommended for repeat micro-TESEs. Some of the patients provided more than one negative micro-TESE history. The remaining nine patients underwent micro-TESE for the first time. They had diagnostic testis spectroscopy prior to the planned micro-TESE. The nine NOA men with a history of previous micro-TESE and the fertile controls underwent MRS following three days of sexual abstinence. The men with NOA were scheduled for micro-TESE after spectroscopy. Detailed information about the surgical technique used for the micro-TESE procedure can be found elsewhere (4,8). The micro-TESE specimens were analyzed by an experienced embryologist to determine whether the materials contained sperm or not. The testis spectroscopy results of the NOA men were analyzed quantitatively and then correlated with the results of subsequent micro-TESE attempts. Possible associations between the metabolite peak intensities obtained from the spectra of the NOA subjects and the sperm retrieval rates in their micro-TESE were assessed. In addition to testis MRS, the testicular long axis and serum concentrations of FSH, luteinizing hormone (LH), prolactin (PRL), and testosterone were measured in each study group. Participants with unilateral testes due to surgical resection or undescended testes were excluded. Subjects with a history of benign or malignant testicular tumors, testicular torsion, and abnormal karyotypes were also not included.

### Magnetic resonance spectroscopy technique

**Figure 1:**
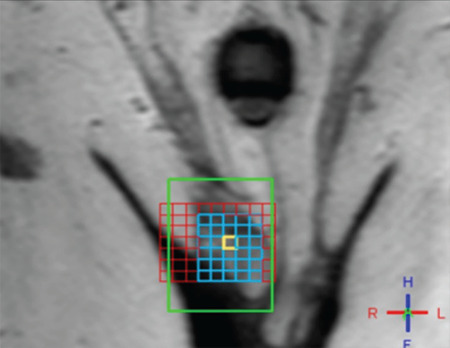
Multivoxel point-resolved spectroscopy sequence was used for detecting testis metabolites. The volume of interest was placed to the center of the testicular parenchyma. Lack of neighbouring organs or tissue parts that could affect signals obtaining from testes make testis spectroscopy easy and objective for obtaining good quality metabolite signals. R: Right, L: Left, H: Head, F: Foot

Both the men with NOA and the fertile controls underwent testis spectroscopy before micro-TESE. Spectroscopy analysis of each testis was performed using a 3-T system (Achieva; Philips, Best, Netherlands). T1-weighted images (WI) [time repetition/time echo (TE), 500/20] and T2-WI (1600/80) with 4 mm thick sections were obtained in the axial and coronal planes. A single and a multi-voxel point-resolved spectroscopy sequence (16), both with short (35 ms) and long (140 ms) TEs were used. Multivoxel point-resolved spectroscopy sequence was used for detecting testes metabolites. The metabolite ratios of the peaks were determined using magnetic resonance user Interface software. The quantified metabolites of the spectra were Cho, Cr, myo-inositol (MI), lactate, and lipids in both NOA groups and the fertile controls. The metabolites in the spectrum were measured in units and converted to parts per million (ppm). The testes were first visualized using magnetic resonance imaging before the voxels were prescribed accordingly (17,18). Due to the critical importance of the voxel locations in the appropriate testicular area for investigating spermatogenesis, the volume of interest was placed to the center of the testicular parenchyma (Figure 1). The absence of neighboring organs or tissue parts that could affect the signals obtained from testes make testis spectroscopy easy and objective, thus resulting in good quality metabolite signals. Possible associations between the metabolite intensities obtained from the spectra of the NOA subjects and the sperm retrieval rates in their micro-TESEs were assessed. The spectroscopy results were also compared with other predictors, including age, FSH, LH, PRL, testosterone, and the long axis of the testes.

### Statistical analysis

SPSS version 23.0 (IBM Corporation, Armonk, NY, USA) was used for statistical analysis of the data. The conformity to normal distribution of the data was tested using the Shapiro-Wilk test. Quantitative data were expressed as mean ± standard deviation, median and range (minimum-maximum), and percentage (%). For comparison of the groups, ANOVA test was used with the corresponding Tukey contrast test. The chi-square or Fisher’s exact tests were performed to compare the frequencies of the categorical variables, as appropriate. The correlations between age, reproductive hormones, and tissue metabolites were evaluated using Pearson correlation coefficients. Receiver operating characteristic curve analysis was used to determine the best cut-off values for the testes metabolites for the evaluation of the success rates of sperm retrieval. Cho and Cr are the two main metabolites indicating the vital function of living cells. In our previous study, it was shown that the metabolic function of reproductive tissues is either absent or pathological when Cho and Cr signals are below the expected physiological values (16). Therefore, these two metabolites were used to determine the cut-off values for the prediction of spermatogenesis in NOA men undergoing micro-TESE. Initially, it was thought that micro-TESE for NOA cases could be recommended where the Cho and Cr signals were greater than the cut-off values. However, due to the novelty of the diagnostic use of spectroscopy in NOA and to determine the cut-off values, micro-TESE was offered for all participants, regardless of their metabolite values. A value of p<0.05 was accepted as statistically significant.

## Results

**Figure 2:**
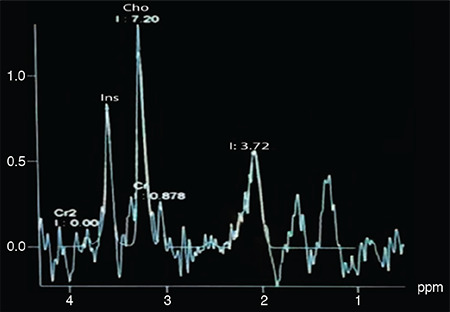
The spectral pattern of a fertile man showing high Cho and MI signals depicting normal Cho: Choline, MI: Myo-inositol, Cr: Creatine

**Figure 3:**
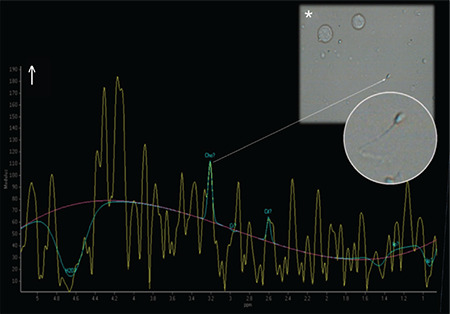
(*) The spectral pattern of an NOA man with positive sperm retrieval following micro-TESE. (↑) Note the high Cho signal depicting active spermatogenesis NOA: Non-obstructive azoospermia, micro-TESE: Micro-dissection testicular sperm extraction, Cho: Choline

**Figure 4:**
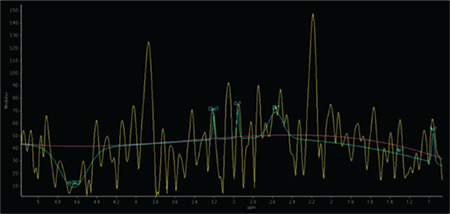
The spectral pattern of an NOA man with negative sperm retrieval following micro-TESE. Note the low Cho and Cr signals depicting pathological spermatogenesis NOA: Non-obstructive azoospermia, micro-TESE: Micro-dissection testicular sperm extraction, Cho: Choline, Cr: Creatine

**Figure 5:**
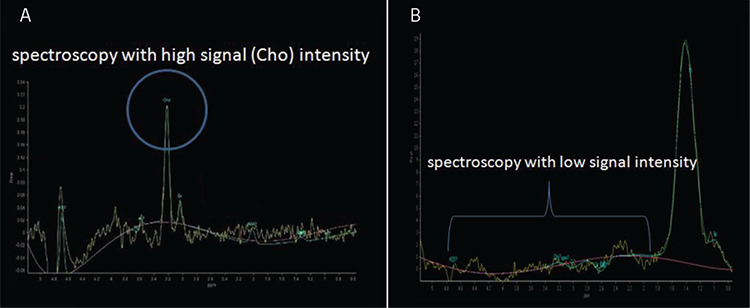
Comparison of a spectroscopy with a high (A) and low (B) signal intensity. It is expected to be a healthy metabolic process in the testis of a man with high Cho peak (A). There is a high probability that the metabolic process is disturbed in the testis of a man with low signal. The first case belongs to a fertile case (A) and the second is a spectroscopy of a TESE negative case (B) Cho: Choline, TESE: Testicular sperm extraction

Demographic characteristics of each group are presented in Table 1. A total of 18 subjects with NOA and five fertile men underwent single/multi-voxel MRS at 3 T. MRS was feasible for in all subjects with NOA, as well as the control subjects. All the patients had two testes; thus, 36 testes were investigated in terms of their peak characteristics. Since the right and left testes signal characteristics were similar, only the right testis data are presented here. As there are no previous studies investigating the effects of spectroscopy on spermatogenesis, all the patients were sent for micro-TESE regardless of their peak intensities. Five different testicular metabolites, including Cho, Cr, Lac, MI, and lipids, were detected via spectroscopy. Cho, Cr, and MI were the most prominent metabolites detected in the fertile group (Table 2 and Figure 2) and this was also the case with the NOA men with active spermatogenesis. The Cho and Cr signals of the fertile group were significantly higher than those in the NOA groups. The MI and lactate metabolites of the fertile group were similar to those of the NOA men with or without sperm in micro-TESE. Although a low lactate signal was detected in the fertile cases compared to the NOA groups, the difference was not statistically significant. When the subgroup analysis was performed, the lactate peak of the NOA men with negative sperm retrieval in micro-TESE was higher than that of the NOA men with positive sperm retrieval (1.515±0.675 ppm vs 0.525±0.193 ppm; p=0.001). Cho, Cr, and MI were highly sensitive peaks to predict the presence of sperm in micro-TESE. The cut-off value of Cho was 1.46 ppm [area under the curve (AUC): 0.938, 95% confidence interval (CI): 0.811-1.00; p=0.002], the cut-off value of Cr was 1.43 ppm (AUC: 0.900, 95% CI: 0.730-1.00; p=0.004), and the cut-off value of MI was 0.79 ppm (AUC: 0.794, 95% CI: 0.547-1.00; p=0.037) for positive sperm retrieval in micro-TESE (Table 3). In five of the nine NOA cases, the Cho and Cr signals were found to be greater than the cut-off values while in the remaining four cases, these were lower than the cut-off values. Sperm was found in four of the five cases with Cho and Cr signals greater than the cut-off values in the initial MRS (Figure 3). In one case, despite high Cho and Cr signals, no sperm was found on micro-TESE. Sperm was not found in three of four cases with Cho and Cr signals lower than the cut-off values in the initial MRS (Figure 4). Despite the low Cho and Cr signals in the initial spectroscopy, sperm was found in one man with NOA. In total, sperm was harvested from five of the nine subjects with NOA during micro-TESE (Table 4). The sperm retrieval rate for the NOA group was 55.5%. A low Cho peak was detected in 100% of the NOA men with negative sperm retrieval in micro-TESE (Figure 5). In contrast, a high Cho peak was detected in 87% of the NOA men with positive sperm retrieval in micro-TESE (Figure 5). A low Cho peak had high specificity thus indicating inactive spermatogenesis. The peak intensities of the measured metabolites in the fertile men were similar to the spectra of the NOA men with sperm in micro-TESE (Cho p=0.059; Cr p=0.917; lactate p=0.530; MI p=0.117; lipid p=0.310). Conversely, the signal characteristics of the fertile men were significantly different than those of the NOA men without sperm in micro-TESE (Cho p=0.001; Cr p=0.017; lactate p=0.002; MI p=0.007). The mean testicular lengths were similar in the fertile and NOA groups. No correlations were detected between the FSH, LH, PRL, and total testosterone levels, long testicular axis, and measured spectral signals (Table 5). However, a significantly positive correlation was detected between age and lactate signal. When the nine men who had a history of previous micro-TESEs were examined retrospectively, the Cho and Cr signals were found to be greater than the cut-off value in three patients with positive sperm retrieval. The Cho and Cr signals were either absent or under the cut-off values in six patients with negative sperm retrieval in previous micro-TESEs.

## Discussion

There is no single clinical or laboratory finding that can accurately predict positive or negative sperm retrieval before micro-TESE. In the present study, the diagnostic accuracy of in vivo spectroscopy signals obtained from the testicles of NOA men undergoing micro-TESE and the concomitant success rates for finding spermatozoa were investigated. The most crucial result of this study was the powerful relationship between a high Cho peak and the chance of sperm retrieval in micro-TESE. An increased Cho signal intensity was very sensitive for predicting positive sperm retrieval when using micro-TESE technique. The chance of sperm retrieval using micro-TESE was very high when the cut-off value for Cho was over 1.46 ppm and the cut-off value for Cr was over 1.43 ppm. Sperm was retrieved by micro-TESE in 80% of the NOA men whose Cho and Cr signals were greater than the cut-off values. Nevertheless, despite high Cho and Cr signals on spectroscopy, sperm could not be detected in one patient. Of the four men with NOA who exhibited a high Cho signal and had successful retrieval of spermatozoa, three of their partners became pregnant. One woman delivered a healthy baby while the remaining two women had ongoing pregnancies during the study period. Accordingly, if the Cho and Cr signals are lower than 1.46 and 1.43 ppm, respectively, the chances of sperm retrieval in micro-TESE are very low. No sperm was found using micro-TESE in 75% of the patients whose Cho and Cr signals were lower than 1 ppm. In fact, 75% of azoospermic patients with low Cho signal did not have any foci of spermatogenesis that were sufficient to find spermatozoa on micro-TESE. Only one NOA man with low Cho signal in prior testis spectroscopy had successful spermatozoa retrieval. This may be due to a technical error in evaluating the spectroscopy signals or a fault in the MRS procedure. Interestingly, his partner did not become pregnant.

Our findings suggest that, irrespective of the overall state of spermatogenesis, determining high Cho and Cr signals may predict positive sperm retrieval in men with NOA. In light of this, we suggest that the best predictor of positive sperm retrieval in micro-TESE is a high Cho peak. A Cho signal at least greater than 1.46 ppm should be present in the MRS of a testicle to find spermatozoa on micro-TESE. Similar to the Cho signal, the Cr signal in the NOA men with active spermatogenesis was found to be greater compared to the Cr signals in the NOA men without spermatogenesis. As Cr is an indicator of the energy status of living cells, a decreased Cr signal in the NOA men without sperm may indicate a defective metabolism within the testis. In contrast to the Cho and Cr signals, the lactate levels were significantly higher in the negative sperm retrieval group when compared to the positive sperm retrieval group (1.515±0.675 vs 0.525±0.193, respectively). As is well known, high lactate levels indicate the presence of anaerobic glycolysis at the cellular level. It is therefore not expected that sperm can survive in an oxygen-free environment. It was observed that the NOA men with active spermatogenesis had high Cho peaks when compared to the NOA men without spermatogenesis. Although the exact mechanism for this difference was unclear, we propose that it may be associated with the disturbed cellular integrity of the Leydig and/or Sertoli cells. The absence of any signal or weak signal intensity in NOA men with Sertoli cells only, maturation arrest, or orchitis support our idea. Albrecht (19) reported that the testes of NOA men showed an increased deposition of collagen fibers and an extracellular matrix. They also noted that by increasing the thickness of the lamina propria, this pathological accumulation may cause defective spermatogenesis. We therefore propose that decreased Cho peak intensity in NOA men without sperm on micro-TESE may be related to excessive thickness of the lamina propria of the seminiferous tubules. The greatest support for our hypothesis comes from the study conducted by Tsili et al. (18), which showed a decline in the intensity of Cho signals with advancing age. When taken together, our findings and previous results suggest that NOA men without active spermatogenesis exhibit the signal properties of elderly men. Conversely, as Cho is a marker of cell membrane turnover, a high Cho peak in NOA men with sperm on micro-TESE may indicate that they have healthy cellular function. A similar Cho peak intensity in fertile men and NOA men with active spermatogenesis further supports our hypothesis. When a routine clinical application of testicular spectroscopy before micro-TESE is possible, this may lead to more cost-effective ICSI cycles because ovarian stimulation will only be started in NOA patients with positive spectroscopy, which predicts the presence of sperm in the testicles. With the use of this non-invasive tool, an infertile man undergoing micro-TESE will know whether their testes contain sperm or not. If the initial micro-TESE is negative for finding sperm, spectroscopy will help in the decision of whether to offer a repeat micro-TESE. If testicular mapping can be performed according to the signal intensities of Cho and Cr, it may help determine in which regions sperm will be found by micro-TESE. Thus, it may be possible to avoid unnecessary surgical procedures in the sperm-free regions of testicular tissue. As a consequence, NOA patients with favorable spectroscopy that predicts the presence of sperm may undergo micro-TESE, confident in the knowledge that sperm will be retrievable during the procedure. In contrast, subjects with unfavorable spectroscopy results can be counseled about the low sperm retrieval rates in micro-TESE. NOA men with unfavorable metabolites at spectroscopy can abstain from micro-TESE attempts and redirect their attention to other assisted reproductive technology options. Although only in a small proportion of NOA patients, spectroscopy can also help detect benign and malignant testicular lesions as well as congenital and acquired causes of obstructive azoospermia (20).

The current investigation was carried out because of two contrasting hypotheses; high Cho and Cr signals were proposed as being indicators of the presence of spermatozoa while low Cho and Cr signals would be indicators of the absence of spermatozoa on micro-TESE. We found that low Cho and Cr signals on spectroscopy indicated that spermatozoa will not be found on micro-TESE while the presence of high Cho and Cr signals in spectroscopy indicated a strong likelihood that sperm will be found in micro-TESE. The real value of prior testis spectroscopy is its ability to correctly predict whether spermatozoa will be present or absent on micro-TESE.

## Conclusion

Analysis of our results demonstrates for the first time that high Cho and Cr signals are the best predictors of positive sperm retrieval in NOA men undergoing micro-TESE. Moreover, MI may also be used as a predictive factor. In addition to evaluating AZF deletions, testicular volume, and serum FSH levels, spectroscopy of the testes before micro-TESE can improve the prediction of sperm retrieval rates in men with azoospermia. Bilateral testicular spectroscopy can not only provide significant information with regard to the possibility of retrieving sperm in micro-TESE, but can also prevent unnecessary surgical interventions. Studies with larger sample sizes are warranted to enable a more adequate assessment of the impacts of in vivo spectroscopy on sperm retrieval rates. If our results are confirmed by other studies, testis spectroscopy could be used in ART practice to distinguish between testes with active or inactive spermatogenesis. In addition to being inexpensive and non-invasive in nature, the quick results of spectroscopy make it an ideal candidate tool for the screening of NOA men before micro-TESE. Testicular MRS is best coupled with an initial micro-TESE before starting the ICSI cycle. This non-invasive technique may serve as a novel and useful predictive method for guiding urologists and IVF specialists on whether to perform or not perform micro-TESE.

## Figures and Tables

**Table 1:**
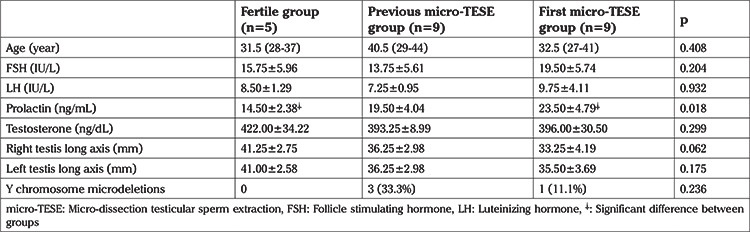
Characteristics of study subjects

**Table 2:**
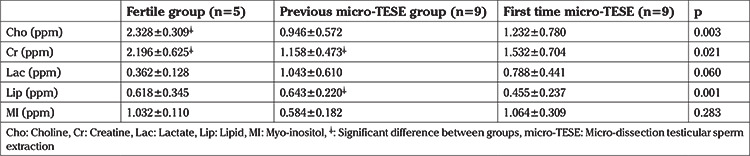
Testis metabolites levels

**Table 3:**
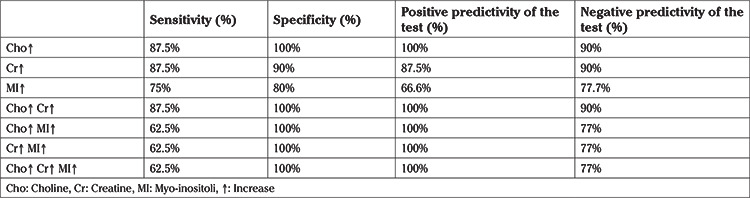
Diagnostic performance of testes metabolite leves for positive sperm retrieval

**Table 4:**
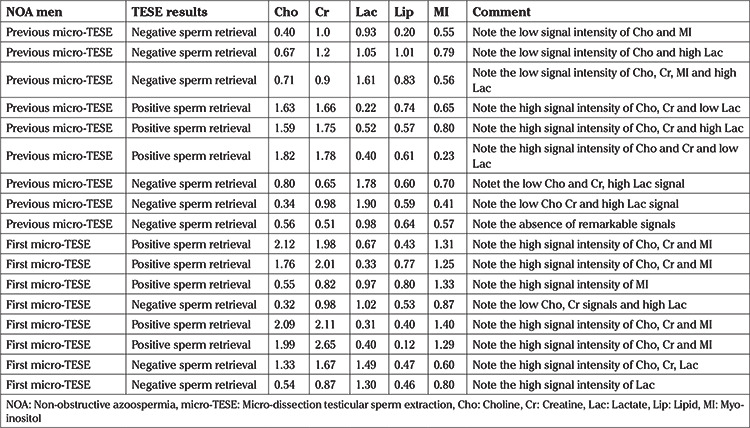
Magnetic resonance spectroscopy analysis of testes of men with negative or positive sperm retrieval in previous or first micro-TESE

**Table 5:**
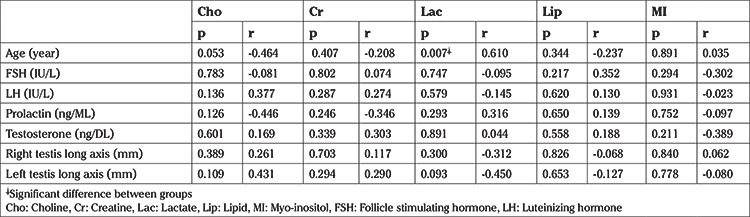
Correlation between age, reproductive hormones and testis metabolites
